# Association between first-trimester serum 25(OH)D and EPDS-positive postpartum depressive symptoms in women with gestational diabetes mellitus: a retrospective cohort study

**DOI:** 10.3389/fpsyt.2026.1886353

**Published:** 2026-09-17

**Authors:** Caijun He, Hongli Zhang, Shuang Han, Liying Zhang, Jinhe Ma, Huizi Tang

**Affiliations:** 1 Department of Obstetrics and Gynaecology, The 962 Hospital, Joint Logistic Support of the Chinese People’s Liberation Army, Harbin, Heilongjiang, China; 2 Nursing Department, The 962 Hospital, Joint Logistic Support of the Chinese People’s Liberation Army, Harbin, Heilongjiang, China

**Keywords:** 25-hydroxyvitamin D, gestational diabetes mellitus, perinatal mental health, postpartum depressive symptoms, pregnancy, retrospective cohort, risk factors, vitamin D

## Abstract

**Introduction:**

Gestational diabetes mellitus (GDM) has been associated with an increased risk of postpartum depressive symptoms, while maternal vitamin D status may also be related to perinatal mood disturbances. This study investigated the association between first-trimester serum 25-hydroxyvitamin D [25(OH)D] concentrations and Edinburgh Postnatal Depression Scale (EPDS)-positive postpartum depressive symptoms in women with GDM.

**Methods:**

This retrospective cohort study included 242 women with GDM. Prenatal depressive and anxiety symptoms were assessed using the Patient Health Questionnaire-9 (PHQ-9) and Generalized Anxiety Disorder-7 (GAD-7), respectively. Sequential logistic regression models were adjusted for baseline maternal characteristics, prenatal PHQ-9 and GAD-7 scores, and glycated hemoglobin A1c (HbA1c).

**Results:**

Among 242 women, 69 had EPDS-positive postpartum depressive symptoms. Each 5-ng/mL increase in 25(OH)D was associated with lower odds of EPDS-positive symptoms in the crude model (OR 0.35, 95% CI 0.25–0.48) and after adjustment for baseline characteristics (adjusted OR 0.36, 95% CI 0.25–0.50). After additional adjustment for prenatal PHQ-9 and GAD-7 scores, the association was attenuated and was no longer statistically significant overall (adjusted OR 0.69, 95% CI 0.43–1.12; P = 0.134). Additional adjustment for HbA1c produced similar results. Exploratory stratified analyses using the full Model 2 covariate set showed no statistically significant associations within strata defined by pre-pregnancy BMI, hypertensive disorders of pregnancy, or insulin use, and no significant interactions were detected.

**Discussion:**

These findings suggest that the overall association between first-trimester 25(OH)D and postpartum depressive symptoms was substantially influenced by prenatal mental health status and was not consistently demonstrated after comprehensive covariate adjustment.

## Introduction

Postpartum depressive symptoms are common and clinically important because they can affect maternal well-being, infant care, mother–infant relationships, and family functioning (1–3). Recognition in routine care can be difficult because fatigue, sleep disturbance, and emotional lability are also common after delivery (1, 4). Identifying women who require closer psychological follow-up therefore remains an important obstetric challenge (2, 3, 5).

Postpartum depressive symptoms arise from interacting biological, psychological, and social factors, including pre-existing emotional vulnerability, pregnancy-related stress, sleep deprivation, adverse obstetric experiences, and maternal comorbidity (2, 3, 6). This multifactorial etiology has motivated investigation of antenatal characteristics associated with postpartum symptoms.

Gestational diabetes mellitus (GDM) is particularly relevant in this context. GDM is one of the most common metabolic complications of pregnancy, and is associated with insulin resistance, increased treatment burden, heightened maternal concern about pregnancy outcomes, and a greater likelihood of obstetrical intervention (7, 8). Beyond its metabolic implications, GDM may be linked to emotional distress during pregnancy and after childbirth. Recently, several clinical analyses have reported an increased risk for postpartum depressive symptoms in women with GDM compared to those without GDM, although the strength of this association varies according to population and study design (7–9). However, some evidence has also indicated that depressive symptoms before and during pregnancy may be linked to subsequent GDM, suggesting that the association between metabolic dysfunction and perinatal mood symptoms may be bidirectional and not strictly unidirectional (9, 10). This overlap highlights the need to evaluate potential correlates of postpartum depressive symptoms within the GDM population while accounting for prenatal psychological status.

As a widely used marker of vitamin D status, serum 25-hydroxyvitamin D [25(OH)D] has been found to be associated with immune regulation and neuroendocrine function (11, 12). Because vitamin D deficiency or insufficiency is prevalent in pregnancy worldwide, maternal low vitamin D status has been examined in relation to several negative outcomes such as altered glucose metabolism, hypertensive disorders, and depressive symptomatology (11–13). Maternal vitamin D deficiency has been associated with postpartum depressive symptoms in recent clinical literature (14). However, the consistency and independence of this association remain uncertain (11–13). Differences in the gestational timing of 25(OH)D measurement, depression-screening instruments and cutoffs, duration of postpartum follow-up, and approaches to confounder adjustment may contribute to the inconsistent findings. Moreover, most previous studies were conducted in general obstetric populations rather than metabolically defined clinical subgroups (11–13). Therefore, despite the growing interest in the potential link between maternal vitamin D status and postpartum depressive symptoms, evidence remains limited regarding whether the association between first-trimester serum 25(OH)D and Edinburgh Postnatal Depression Scale (EPDS)-positive postpartum depressive symptoms persists after accounting for prenatal depressive and anxiety symptoms in women with GDM.

Therefore, this retrospective cohort study examined the association between first-trimester serum 25(OH)D and EPDS-positive postpartum depressive symptoms in women with GDM. We further assessed the extent to which this association changed after adjustment for baseline maternal characteristics, prenatal depressive and anxiety symptoms, and glycemic status. Additional sensitivity and exploratory analyses were performed to evaluate the consistency and functional form of the observed association.

## Materials and methods

### Study design, population, and setting

This study was determined to be exempt from ethics review and approval by the Medical Ethics Committee of the 962nd Hospital of the Joint Logistics Support Force of the Chinese People’s Liberation Army because it used only anonymized retrospective clinical data and involved no research-specific intervention or specimen collection. Written informed consent was therefore not required.

Postpartum follow-up and data extraction were completed by 30 June 2026, allowing all included women to complete the scheduled postpartum EPDS assessment. During the study period, first-trimester serum 25(OH)D testing, Patient Health Questionnaire-9 (PHQ-9) and Generalized Anxiety Disorder-7 (GAD-7) assessments were components of routine antenatal care, and Edinburgh Postnatal Depression Scale (EPDS) screening was routinely performed at the postpartum follow-up visit.

### Inclusion/exclusion criteria

The inclusion criteria were as follows: delivery at the study hospital during the predefined study period; diagnosis of GDM during the index pregnancy according to the specified diagnostic criteria; availability of a first-trimester serum 25(OH)D measurement; availability of prenatal PHQ-9 and GAD-7 scores; and completion of postpartum EPDS assessment. The exclusion criteria were as follows: multiple pregnancy, pregestational diabetes, documented major psychiatric disorders before pregnancy, or missing key clinical information.

Of the 263 women screened, 21 were excluded because of the absence of first-trimester 25(OH)D data (n = 3), absence of postpartum EPDS data (n = 4), multiple pregnancy (n = 2), pregestational diabetes (n = 4), documented major psychiatric disorders before pregnancy (n = 5), or missing key clinical information (n = 3). Because the screening log for excluded women contained eligibility and exclusion information but did not contain complete baseline clinical variables, a formal comparison between included and excluded women could not be performed. The participant selection process is shown in **Figure 1**.

**Figure 1:**
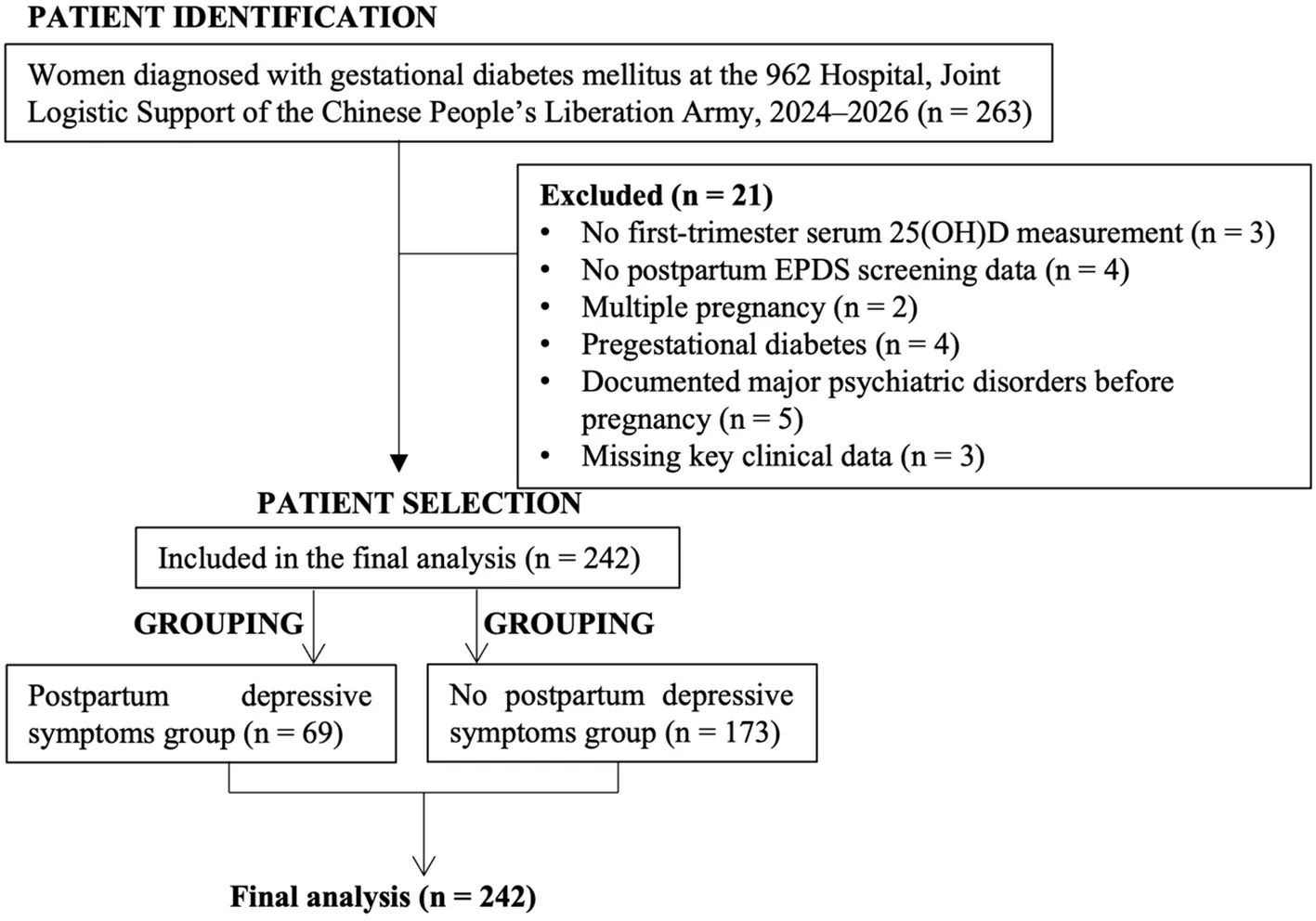
Flow diagram of patient selection. 25(OH)D, 25-hydroxyvitamin D; EPDS, edinburgh postnatal depression scale.

### Diagnosis of GDM

GDM was diagnosed using a one-step 75-g, 2-hour oral glucose tolerance test performed at 24–28 weeks of gestation. The diagnostic thresholds were fasting plasma glucose ≥ 5.1 mmol/L, 1-hour plasma glucose ≥ 10.0 mmol/L, or 2-hour plasma glucose ≥ 8.5 mmol/L, with one or more abnormal values considered sufficient for diagnosis (15). The gestational age at GDM diagnosis was recorded for each participant. In addition, glycated hemoglobin A1c (HbA1c) was used as an indicator of glycemic control. Fasting plasma glucose, OGTT glucose values, and HbA1c levels were analyzed in the hospital laboratory using standard automated biochemical methods according to the manufacturer’s instructions.

### Assessment of first-trimester vitamin D status

The main exposure variable was first-trimester serum 25-hydroxyvitamin D [25(OH)D]. Blood samples were collected during the first trimester, and gestational age at sampling was recorded for each participant. Serum 25(OH)D was measured using the ARCHITECT 25-OH Vitamin D assay (Abbott Laboratories, Abbott Park, IL, USA) on an ARCHITECT i2000SR immunoassay analyzer (Abbott Laboratories), according to the manufacturer’s instructions. It was analyzed as a continuous exposure and scaled per 5-ng/mL increase to facilitate clinical interpretation.

### Assessment of prenatal mental health

Prenatal depressive and anxiety symptoms were assessed during early pregnancy using the validated Chinese versions of the Patient Health Questionnaire-9 (PHQ-9) and Generalized Anxiety Disorder-7 (GAD-7), respectively (16, 17). Assessments were performed between 8.0 and 13.9 weeks of gestation. PHQ-9 and GAD-7 total scores were analyzed as continuous covariates. Information regarding psychiatric consultation and antidepressant use during pregnancy was also extracted from the clinical records.

### Assessment of postpartum depressive symptoms

The primary outcome was EPDS-positive postpartum depressive symptoms. Postpartum symptoms were assessed using the validated Chinese-language version of the Edinburgh Postnatal Depression Scale (EPDS) between 35 and 52 days after delivery (42.07 ± 4.01 days). The questionnaire was self-administered by 218 women and completed with staff assistance by 24 women; all 242 questionnaires contained responses to all 10 items. An EPDS score ≥10 was used as the primary threshold, corresponding to the validated 9/10 cutoff reported in Chinese postpartum women (18). Because the Mainland Chinese validation study supported a cutoff greater than 10, a threshold of ≥11 was examined in sensitivity analysis (19); ≥13 and the continuous EPDS score were also evaluated. EPDS item 10 assesses thoughts of self-harm during the preceding 7 days. Any non-zero response was recorded as item 10 positivity but was not used independently to define the primary outcome. According to the institutional clinical pathway, any non-zero response to EPDS item 10 was intended to prompt immediate clinical review and suicide-risk assessment, with psychiatric referral when indicated. However, information on the actual downstream assessment, referral, and management of individual women was not extracted for the present analysis.

### Subgroup and sensitivity analyses

Sensitivity analyses were performed using Firth penalized logistic regression to reduce small-sample and separation bias. The outcome was redefined using EPDS thresholds of ≥11 and ≥13, and the continuous EPDS total score was analyzed as an additional outcome. Further analyses excluded women with psychiatric consultation or antidepressant use during pregnancy. Insulin use, hypertensive disorders of pregnancy, anemia, sleep disorder, and vitamin D supplementation were evaluated individually as post-exposure pregnancy-related factors rather than included simultaneously in the primary adjusted model. Influence analyses were conducted by excluding observations with Cook’s distance >4/n and, separately, serum 25(OH)D values below the 1st or above the 99th percentile. Exploratory subgroup analyses were conducted according to pre-pregnancy BMI (<24 vs ≥24 kg/m^2^) (20), hypertensive disorders of pregnancy, and insulin use. All stratified analyses were performed using Firth logistic regression with the full Model 2 covariate set, including maternal age, pre-pregnancy BMI as a continuous variable, primiparity, vitamin D sampling season, employment status, prenatal PHQ-9 score, and prenatal GAD-7 score. Pre-pregnancy BMI was retained as a continuous covariate even in the BMI-stratified analyses. All subgroup findings were considered exploratory.

### Statistical analysis

In this study, statistical analyses were performed using IBM SPSS Statistics for Windows, version 26.0 (IBM Corporation, Armonk, NY, USA). All statistical tests were two-sided, and P < 0.05 was considered statistically significant. Continuous variables were summarized as means and standard deviations and compared using independent-samples t tests. Categorical variables were summarized as numbers and percentages and compared using the chi-square test or Fisher’s exact test. No missing values were present in the variables included in the reported analyses.

The study size was determined by the number of eligible women available within the prespecified study period. All women who delivered at the study hospital between 1 April 2024 and 1 April 2026 and met the eligibility criteria were included; therefore, no additional sampling was performed. Meanwhile, the multivariable models were kept parsimonious, and effect estimates are reported with 95% confidence intervals to indicate statistical precision.

Covariates were selected based on temporal precedence and previously reported associations with vitamin D status or postpartum depressive symptoms. Variables determined before or at the time of first-trimester 25(OH)D measurement were considered potential baseline confounders. Variables arising later in pregnancy were treated as post-exposure factors and were examined only in exploratory analyses to reduce the risk of overadjustment. Employment status was included as the socioeconomic indicator to maintain model parsimony.

Model 1 was adjusted for maternal age, pre-pregnancy BMI, primiparity, season of blood sampling, and employment status. Model 2 was additionally adjusted for prenatal PHQ-9 and GAD-7 scores. Model 3 was further adjusted for HbA1c as an exploratory glycemic-status model. Post-exposure pregnancy-related factors, including insulin use and hypertensive disorders of pregnancy, were evaluated separately in exploratory analyses. Percentage attenuation after adjustment for prenatal PHQ-9 and GAD-7 scores was calculated as 100 × [1 − |βModel 2|/|βModel 1|], where β represents the regression coefficient for first-trimester serum 25(OH)D.

Firth penalized logistic regression was performed as a sensitivity analysis to reduce small-sample bias and address potential separation (21). Alternative EPDS thresholds of ≥11 and ≥13 were analyzed using Firth logistic regression. The continuous EPDS score was analyzed using linear regression with HC3 heteroscedasticity-consistent standard errors.

Restricted cubic spline analysis was used to examine the functional form of the association between serum 25(OH)D and EPDS-positive postpartum depressive symptoms (22). Four knots were placed at 11.1, 18.7, 23.3, and 30.8 ng/mL, with 20 ng/mL as the reference value. The overall association was tested jointly for all spline terms, whereas non-linearity was tested jointly for the nonlinear spline terms.

Influential observations were evaluated using Cook’s distance and DFBETA. Multicollinearity was assessed using variance inflation factors, and model fit was evaluated using the Hosmer–Lemeshow goodness-of-fit test.

## Results

### Baseline characteristics

Baseline and clinical characteristics of the 242 women are presented in **Table 1**. Women with EPDS-positive postpartum depressive symptoms had lower first-trimester serum 25(OH)D levels than women without EPDS-positive symptoms (P < 0.001). They also had higher pre-pregnancy BMI, fasting plasma glucose, 2-hour OGTT glucose, HbA1c, prenatal PHQ-9 scores, and prenatal GAD-7 scores. Insulin use, hypertensive disorders of pregnancy, anemia, and sleep disorder were more frequent in women with EPDS-positive symptoms, whereas employment was less frequent. Psychiatric consultation and antidepressant use during pregnancy were uncommon and did not differ significantly between groups. Five women (2.1%) had a non-zero response to EPDS item 10, including three women with a score of 1 and two women with a score of 2. All five women had an EPDS total score ≥10.

**Table 1:** Characteristics of women with gestational diabetes mellitus according to EPDS-positive postpartum depressive symptom status.

Variable	EPDS-positive symptoms (n = 69)	No EPDS-positive symptoms (n = 173)	P value
Baseline and early-pregnancy characteristics			
Age, years	31.49 ± 4.41	30.73 ± 3.98	0.214
Prepregnancy BMI, kg/m²	27.24 ± 3.23	25.34 ± 3.00	<0.001
Primipara	38 (55.1%)	84 (48.6%)	0.360
First-trimester serum 25(OH)D, ng/mL	16.47 ± 4.89	22.62 ± 5.61	<0.001
Gestational age at 25(OH)D sampling, weeks	10.63 ± 1.26	10.46 ± 1.19	0.329
Vitamin D sampling season			0.771
Spring	17 (24.6%)	42 (24.3%)	
Summer	17 (24.6%)	42 (24.3%)	
Autumn	14 (20.3%)	45 (26.0%)	
Winter	21 (30.4%)	44 (25.4%)	
Education level			0.004
Low	24 (34.8%)	27 (15.6%)	
Middle	25 (36.2%)	79 (45.7%)	
High	20 (29.0%)	67 (38.7%)	
Employed	39 (56.5%)	126 (72.8%)	0.014
Prenatal PHQ-9 score	5.36 ± 1.65	2.14 ± 1.89	<0.001
Prenatal GAD-7 score	4.70 ± 2.05	2.05 ± 1.72	<0.001
Psychiatric consultation during pregnancy	2 (2.9%)	4 (2.3%)	1.000
Antidepressant use during pregnancy	0 (0.0%)	1 (0.6%)	1.000
Glycemic and post-exposure pregnancy characteristics			
Gestational age at GDM diagnosis, weeks	25.70 ± 1.08	25.91 ± 1.10	0.180
Fasting plasma glucose, mmol/L	5.59 ± 0.53	5.37 ± 0.42	0.004
OGTT 1-hour glucose, mmol/L	10.71 ± 1.19	10.44 ± 0.96	0.093
OGTT 2-hour glucose, mmol/L	9.05 ± 0.96	8.53 ± 0.92	<0.001
HbA1c, %	5.83 ± 0.41	5.54 ± 0.35	<0.001
Insulin use during pregnancy	33 (47.8%)	43 (24.9%)	<0.001
Hypertensive disorders of pregnancy	21 (30.4%)	23 (13.3%)	0.002
Anemia	23 (33.3%)	35 (20.2%)	0.031
Sleep disorder	26 (37.7%)	24 (13.9%)	<0.001
Vitamin D supplementation during pregnancy	33 (47.8%)	88 (50.9%)	0.669
Outcome assessment			
Postpartum days at EPDS assessment	42.65 ± 4.37	41.83 ± 3.85	0.176

Data are presented as mean ± standard deviation or n (%). EPDS, Edinburgh Postnatal Depression Scale; BMI, body mass index; 25(OH)D, 25-hydroxyvitamin D; PHQ-9, Patient Health Questionnaire-9; GAD-7, Generalized Anxiety Disorder-7; GDM, gestational diabetes mellitus; OGTT, oral glucose tolerance test; HbA1c, glycated hemoglobin A1c.

### Sequential regression analyses

The results of the sequential regression analyses are presented in **Table 2** and **Figure 2**. In the crude analysis, each 5-ng/mL increase in first-trimester serum 25(OH)D was associated with lower odds of EPDS-positive postpartum depressive symptoms (OR 0.35, 95% CI 0.25–0.48; P < 0.001). The association remained significant after adjustment for age, pre-pregnancy BMI, primiparity, vitamin D sampling season, and employment status (adjusted OR 0.36, 95% CI 0.25–0.50; P < 0.001).

**Figure 2:**
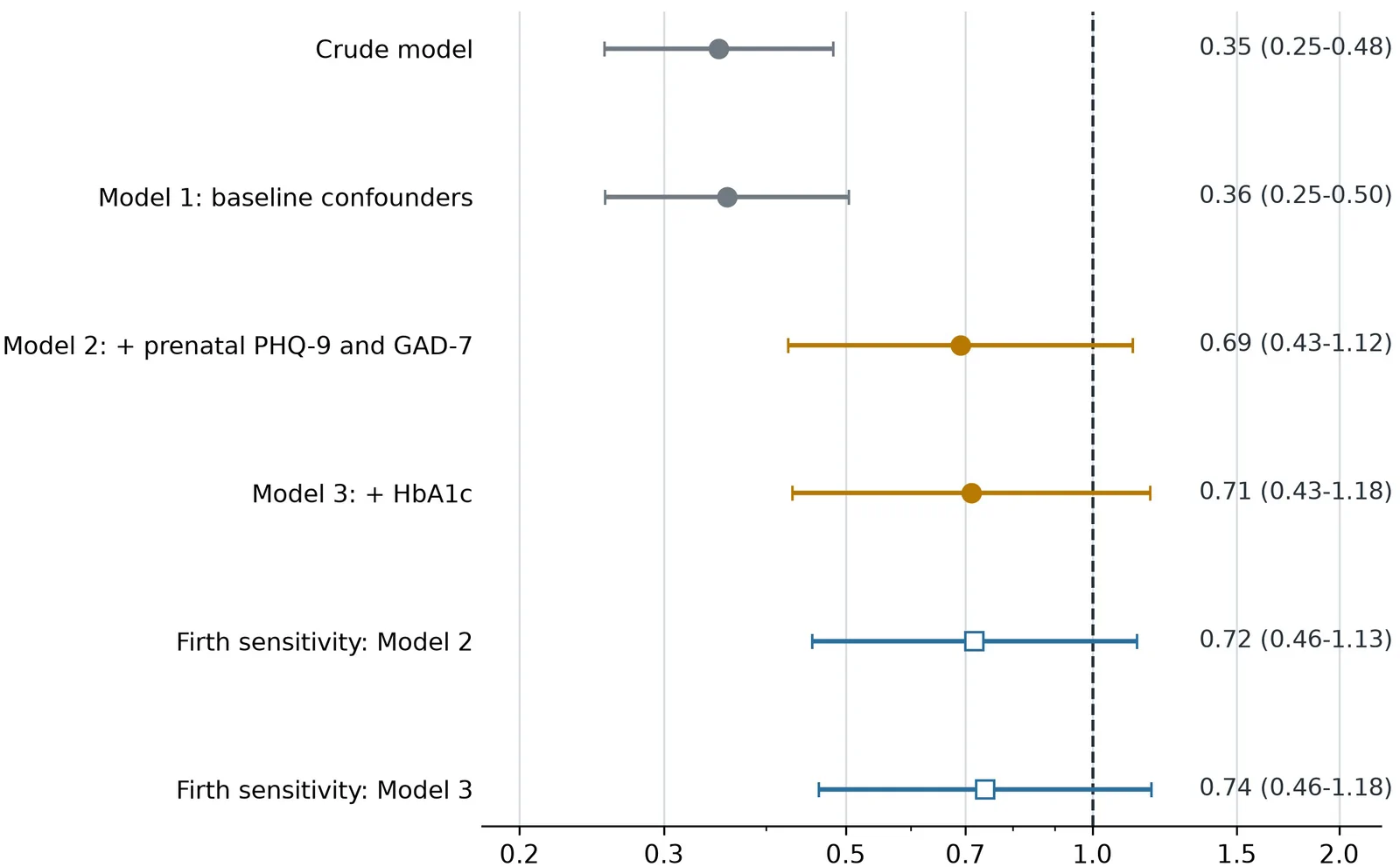
Sequential estimates for the association between first-trimester serum 25(OH)D and EPDS-positive postpartum depressive symptoms. Points show odds ratios per 5 ng/mL higher 25(OH)D; horizontal lines show 95% confidence intervals. 25(OH)D, 25-hydroxyvitamin D; EPDS, Edinburgh Postnatal Depression Scale; PHQ-9, Patient Health Questionnaire-9; GAD-7, Generalized Anxiety Disorder-7; HbA1c, glycated hemoglobin A1c.

**Table 2:** Association between first-trimester serum 25(OH)D and EPDS-positive postpartum depressive symptoms across sequential models.

Model	Adjustment set	OR per 5 ng/mL (95% CI)	P value	Change vs model 1
Crude model	None	0.35 (0.25–0.48)	<0.001	
Model 1: baseline confounders	Age, prepregnancy BMI, primiparity, vitamin D sampling season, and employment	0.36 (0.25–0.50)	<0.001	Reference
Model 2: prenatal mental-health-adjusted model	Model 1 + prenatal PHQ-9 and GAD-7	0.69 (0.43–1.12)	0.134	64.0% attenuation
Model 3: glycemic extension	Model 2 + HbA1c	0.71 (0.43–1.18)	0.185	66.9% attenuation
Firth sensitivity: Model 2	Firth penalized logistic regression using Model 2 covariates	0.72 (0.46–1.13)	0.155	
Firth sensitivity: Model 3	Firth penalized logistic regression using Model 3 covariates	0.74 (0.46–1.18)	0.206	

Odds ratios are reported per 5 ng/mL higher first-trimester serum 25(OH)D. Maximum variance inflation factor was 1.56; Hosmer–Lemeshow P values were 0.502 for Model 2 and 0.911 for Model 3. 25(OH)D, 25-hydroxyvitamin D; OR, odds ratio; CI, confidence interval; BMI, body mass index; PHQ-9, Patient Health Questionnaire-9; GAD-7, Generalized Anxiety Disorder-7; HbA1c, glycated hemoglobin A1c. The primary outcome was EPDS-positive postpartum depressive symptoms, defined as an EPDS score ≥10.

After prenatal PHQ-9 and GAD-7 scores were added, the 25(OH)D regression coefficient was attenuated by 64.0%, and the association was no longer statistically significant (adjusted OR 0.69, 95% CI 0.43–1.12; P = 0.134). Additional adjustment for HbA1c produced a similar estimate (adjusted OR 0.71, 95% CI 0.43–1.18; P = 0.185). Firth penalized logistic regression yielded comparable results for Model 2 (adjusted OR 0.72, 95% CI 0.46–1.13; P = 0.155) and Model 3 (adjusted OR 0.74, 95% CI 0.46–1.18; P = 0.206).

### Sensitivity analyses

Results of the robustness analyses are presented in **Table 3**. The association remained non-significant when EPDS thresholds of ≥11 and ≥13 were used and when EPDS total score was analyzed as a continuous outcome. Exclusion of women with psychiatric consultation or antidepressant use during pregnancy did not materially alter the estimate. Individual adjustment for insulin use, hypertensive disorders of pregnancy, anemia, sleep disorder, or vitamin D supplementation also yielded non-significant estimates.

**Table 3:** Robustness analyses of the association between first-trimester serum 25(OH)D and postpartum depressive symptoms.

Analysis type	Specification	N	Events	Estimate (95% CI)	P value
Alternative outcome definition	EPDS ≥ 10	242	69	0.72 (0.46–1.13)	0.155
Alternative outcome definition	EPDS ≥ 11	242	61	0.76 (0.50–1.17)	0.213
Alternative outcome definition	EPDS ≥ 13	242	50	0.84 (0.54–1.31)	0.448
Alternative outcome definition	Continuous EPDS score	242		-0.36 (-0.96–0.25)	0.250
Psychiatric-record exclusion	Excluded psychiatric consultation or antidepressant use during pregnancy	235	67	0.79 (0.49–1.25)	0.311
Post-exposure exploratory adjustment	Additional adjustment for insulin use	242	69	0.72 (0.45–1.15)	0.171
Post-exposure exploratory adjustment	Additional adjustment for hypertensive disorders of pregnancy	242	69	0.71 (0.44–1.14)	0.157
Post-exposure exploratory adjustment	Additional adjustment for anemia	242	69	0.77 (0.48–1.23)	0.269
Post-exposure exploratory adjustment	Additional adjustment for sleep disorder	242	69	0.75 (0.47–1.20)	0.235
Post-exposure exploratory adjustment	Additional adjustment for vitamin D supplementation	242	69	0.73 (0.46–1.18)	0.202
Influence/extreme-value sensitivity	Excluding observations with Cook’s distance >4/n	217	53	0.19 (0.04–0.93)	0.041
Influence/extreme-value sensitivity	Excluding 1st and 99th percentile tails of 25(OH)D	236	66	0.73 (0.46–1.15)	0.174

Binary outcomes were analyzed using Firth penalized logistic regression with the Model 2 covariates. Continuous EPDS score was analyzed using linear regression with HC3 robust standard errors; this row reports the mean score difference, whereas all other rows report odds ratios per 5 ng/mL higher 25(OH)D. Post-exposure factors were added one at a time. Maximum Cook’s distance was 0.073 and the maximum absolute DFBETA for 25(OH)D was 0.411. 25(OH)D, 25-hydroxyvitamin D; EPDS, Edinburgh Postnatal Depression Scale; CI, confidence interval; HC3, heteroscedasticity-consistent type 3.

Influence diagnostics identified no observation with Cook’s distance >1, although 25 observations exceeded the 4/n threshold. Excluding these observations produced a stronger estimate (adjusted OR 0.19, 95% CI 0.04–0.93; P = 0.041), whereas exclusion of serum 25(OH)D values below the 1st or above the 99th percentile yielded a non-significant result (adjusted OR 0.73, 95% CI 0.46–1.15; P = 0.174).

### Graphical presentation of the association

Restricted cubic spline analysis showed no statistically significant overall association between first-trimester serum 25(OH)D and EPDS-positive postpartum depressive symptoms after adjustment (P for overall association = 0.477), and there was no evidence of non-linearity (P for non-linearity = 0.912). Estimates at clinically relevant concentrations showed an inverse direction, but all confidence intervals were wide and included the null value (**Table 4**; **Figure 3**).

**Table 4:** Restricted cubic spline estimates at clinically relevant first-trimester serum 25(OH)D concentrations.

25(OH)D, ng/mL	Reference, ng/mL	Adjusted OR (95% CI)
10	20	1.80 (0.30–10.77)
15	20	1.34 (0.71–2.56)
20	20	1.00 (1.00–1.00)
25	20	0.75 (0.31–1.83)
30	20	0.58 (0.07–4.51)

Four knots were placed at 11.1, 18.7, 23.3, and 30.8 ng/mL, with 20 ng/mL as the reference. P for overall association = 0.477; P for nonlinearity = 0.912. The prenatal mental-health-adjusted model (Model 2) was used. 25(OH)D, 25-hydroxyvitamin D; OR, odds ratio; CI, confidence interval.

**Figure 3:**
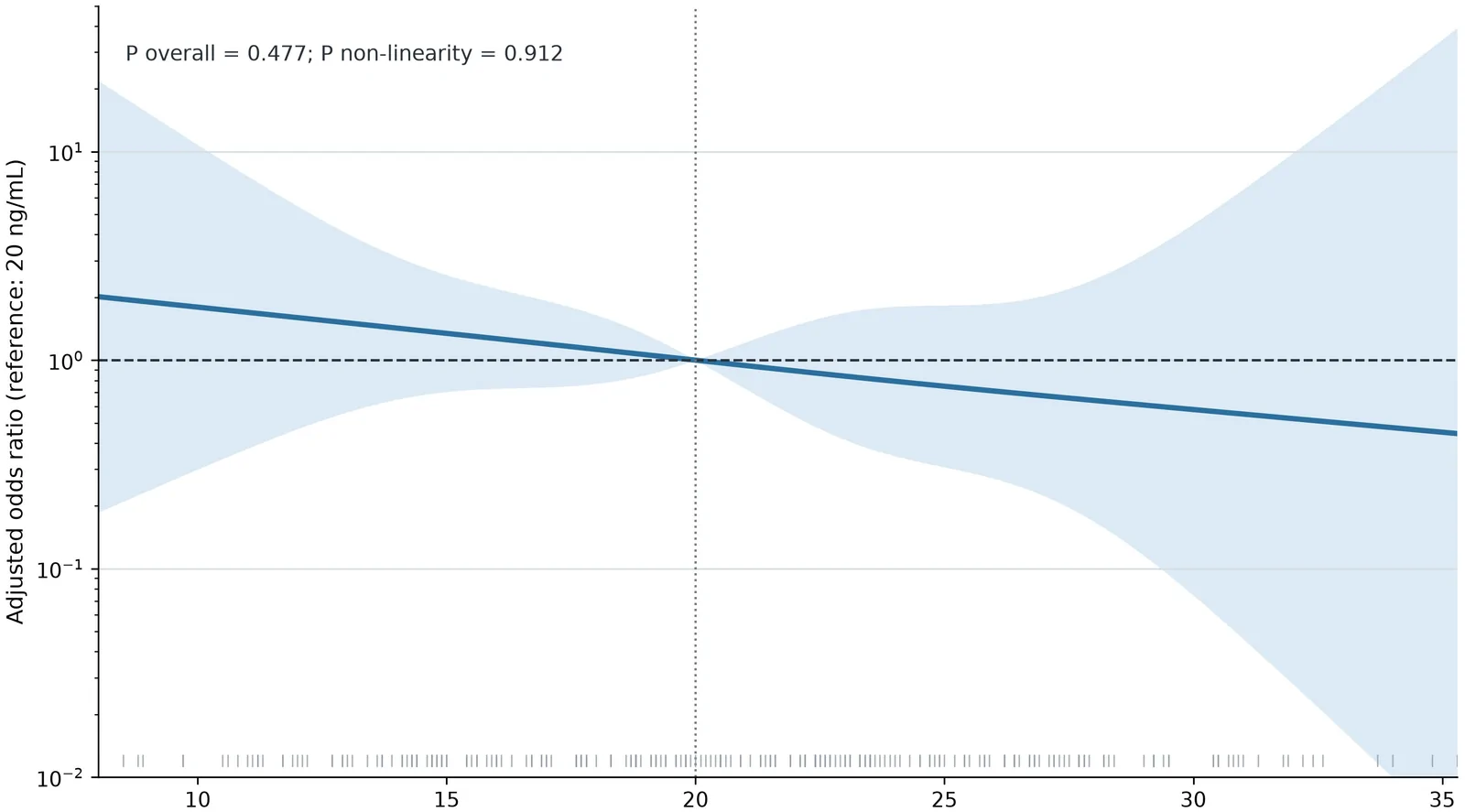
Restricted cubic spline analysis of first-trimester serum 25(OH)D and EPDS-positive postpartum depressive symptoms. The curve was adjusted using the primary adjusted model. The shaded area shows the 95% confidence interval, rug marks show the observed 25(OH)D distribution, and 20 ng/mL was the reference. 25(OH)D, 25-hydroxyvitamin D; EPDS, Edinburgh Postnatal Depression Scale; PHQ-9, Patient Health Questionnaire-9; GAD-7, Generalized Anxiety Disorder-7; HbA1c, glycated hemoglobin A1c.

### Exploratory subgroup analyses

Exploratory subgroup analyses are presented in **Table 5** and **Figure 4**. After adjustment using the full Model 2 covariate set, no statistically significant association between first-trimester serum 25(OH)D and EPDS-positive postpartum depressive symptoms was observed in any of the examined strata. The estimate was inverse among women with pre-pregnancy BMI ≥24 kg/m^2^ (adjusted OR 0.60, 95% CI 0.35–1.01; P = 0.054), whereas the point estimate was above 1 among women with BMI <24 kg/m² (adjusted OR 1.36, 95% CI 0.52–3.53; P = 0.530). Estimates were also non-significant in strata defined by hypertensive disorders of pregnancy and insulin use. No statistically significant interactions were observed for BMI (P for interaction = 0.217), hypertensive disorders of pregnancy (P for interaction = 0.477), or insulin use (P for interaction = 0.702).

**Table 5:** Exploratory subgroup analyses of first-trimester serum 25(OH)D and EPDS-positive postpartum depressive symptoms.

Subgroup	Stratum	N	Events	Adjusted OR per 5 ng/mL (95% CI)	P value	P for interaction
Prepregnancy BMI	BMI <24 kg/m²	65	10	1.36 (0.52–3.53)	0.53	0.217
Prepregnancy BMI	BMI ≥24 kg/m²	177	59	0.60 (0.35–1.01)	0.054	0.217
HDP	No HDP	198	48	0.59 (0.34–1.02)	0.06	0.477
HDP	With HDP	44	21	0.68 (0.20–2.30)	0.54	0.477
Insulin use	No insulin use	166	36	0.65 (0.36–1.17)	0.154	0.702
Insulin use	With insulin use	76	33	0.69 (0.32–1.50)	0.347	0.702

Exploratory estimates were obtained using Firth logistic regression with the full Model 2 covariate set, including maternal age, pre-pregnancy BMI as a continuous variable, primiparity, vitamin D sampling season, employment status, prenatal PHQ-9 score, and prenatal GAD-7 score. Pre-pregnancy BMI was retained as a continuous covariate in the BMI-stratified analyses. The primary outcome was EPDS-positive postpartum depressive symptoms, defined as an EPDS score ≥10.

**Figure 4:**
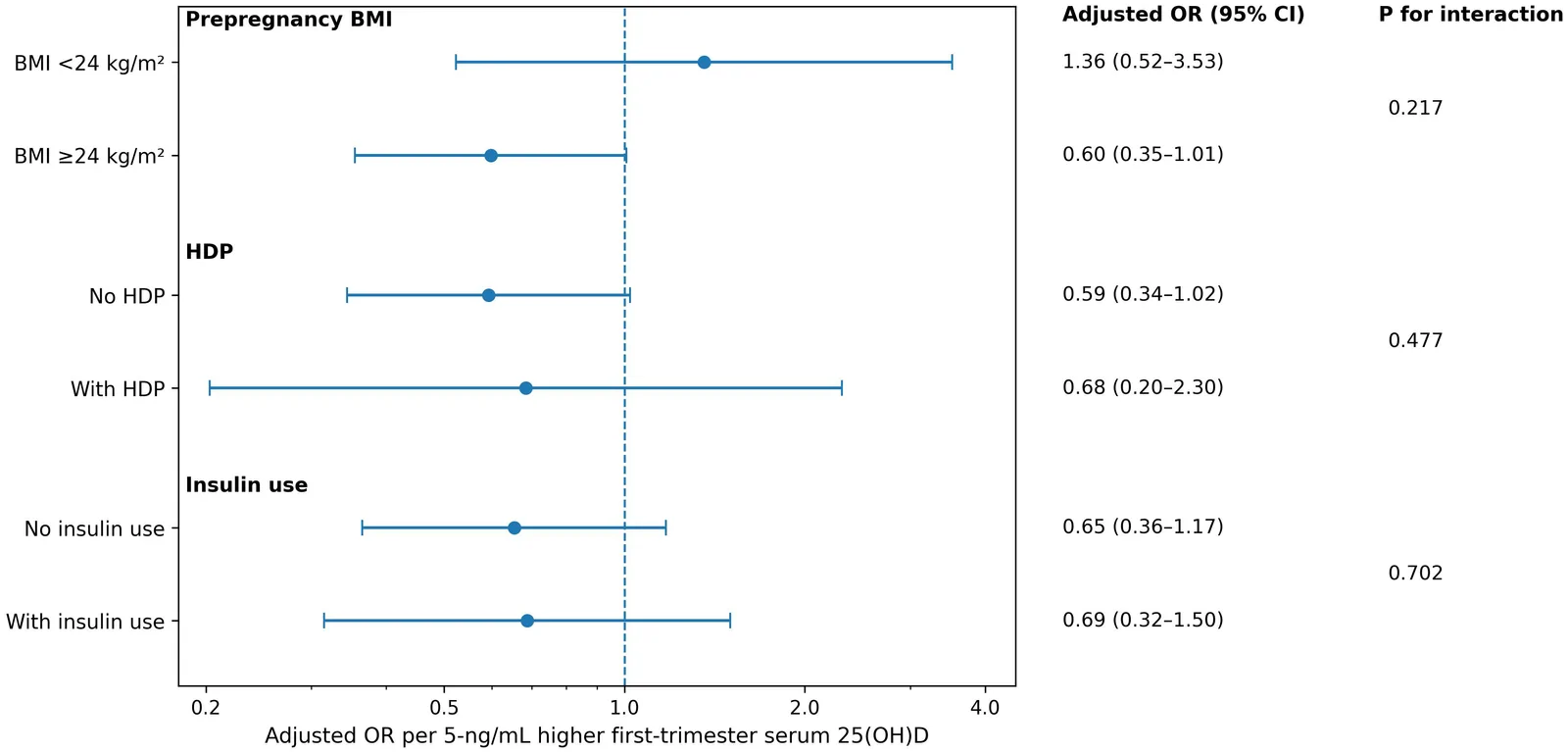
Exploratory subgroup analyses. Points show Firth-adjusted odds ratios per 5 ng/mL higher 25(OH)D; horizontal lines show 95% confidence intervals. 25(OH)D, 25-hydroxyvitamin D; EPDS, Edinburgh Postnatal Depression Scale; BMI, body mass index; HDP, hypertensive disorders of pregnancy; OR, odds ratio; CI, confidence interval.

## Discussion

In this retrospective cohort study of women with GDM, lower first-trimester serum 25(OH)D was strongly associated with EPDS-positive postpartum depressive symptoms in the crude and baseline-adjusted analyses. However, after prenatal PHQ-9 and GAD-7 scores were included, the 25(OH)D regression coefficient was attenuated by 64.0%, and the association was no longer statistically significant. Additional adjustment for HbA1c and analysis using Firth penalized logistic regression produced similar non-significant estimates. Therefore, the present findings do not support an association between first-trimester serum 25(OH)D and postpartum depressive symptoms that is independent of prenatal mental-health status.

An updated meta-analysis reported that women with GDM have a higher probability of postpartum depression or depressive symptoms than women without GDM, although substantial heterogeneity exists across studies (23). A prospective cohort study similarly found an association between GDM and postpartum depressive symptomatology but emphasized the importance of maternal and pregnancy-related covariates (10). The present study differs from these investigations because all participants had GDM; therefore, our analysis focused on factors associated with variation in postpartum depressive symptoms within an already metabolically vulnerable population.

Maternal vitamin D status has also been investigated in relation to perinatal depressive symptoms. A meta-analysis of longitudinal studies reported an inverse association between vitamin D status and antenatal or postpartum depression (12). However, a broader systematic review found that results varied according to study design, timing of vitamin D measurement, depressive symptom assessment, and confounder adjustment (24). Our findings may help explain part of this inconsistency. The association was strong before prenatal psychological status was considered but substantially weaker after prenatal PHQ-9 and GAD-7 scores were included.

The attenuation following adjustment for prenatal psychological symptoms is clinically and methodologically important. Prenatal maternal depression has previously been associated with postpartum depression among women with GDM (25). Depressive and anxiety symptoms during pregnancy may also influence dietary intake, outdoor activity, sleep, physical activity, and supplement use, all of which may be related to vitamin D status. Furthermore, the relationship between metabolic abnormalities and perinatal depressive symptoms may be bidirectional rather than unidirectional (26). Because prenatal PHQ-9, GAD-7, and serum 25(OH)D were assessed during the same early-pregnancy period, the present retrospective study cannot determine whether prenatal psychological symptoms represent confounders, early manifestations of a continuing perinatal mood process, or parallel consequences of shared determinants. Nevertheless, the observed attenuation demonstrates that prenatal mental health status should be considered when evaluating the association between vitamin D and postpartum depressive symptoms.

Several biological pathways have been proposed to connect vitamin D status with depressive symptoms, including inflammatory regulation, neuroendocrine activity, and serotonergic function (11). Vitamin D deficiency has also been linked to impaired glucose metabolism and the development of GDM, suggesting possible overlap between metabolic and psychological pathways (27). However, the current observational results do not establish any of these mechanisms. Women with EPDS-positive postpartum depressive symptoms had higher pre-pregnancy BMI, less favorable glycemic measures, and higher frequencies of insulin use, hypertensive disorders of pregnancy, anemia, and sleep disorder. These findings indicate a greater overall clinical burden but should not be interpreted as evidence that these factors caused postpartum depressive symptoms.

Exploratory adjustment for post-exposure pregnancy factors, including insulin use, hypertensive disorders of pregnancy, anemia, sleep disorder, and vitamin D supplementation, did not materially change the non-significant estimate obtained after adjustment for prenatal PHQ-9 and GAD-7 scores. Analyses using alternative EPDS thresholds, the continuous EPDS score, and restricted cubic splines likewise did not provide consistent evidence of an independent association. However, exclusion of observations exceeding the Cook’s distance screening threshold changed the statistical significance of the estimate, indicating sensitivity to selected influential observations.

The fully adjusted stratified logistic regression analyses did not provide evidence of statistically significant associations within any of the examined subgroups, and none of the interaction tests was significant. However, among women with pre-pregnancy BMI ≥24 kg/m², the association was of borderline statistical significance (P = 0.054), suggesting that in overweight or obese women with GDM, the association between vitamin D and EPDS-positive postpartum depressive symptoms could be independent of both prenatal mental health and pre-pregnancy BMI. This finding warrants further investigation in this subgroup. The proportion of women with EPDS-positive postpartum depressive symptoms was approximately twice as high in this subgroup as in women with BMI <24 kg/m^2^ (33.3% vs 15.4%). Moreover, statistical power was limited in some strata, particularly among women with BMI <24 kg/m^2^, in whom only 10 EPDS-positive events were observed, and among women with hypertensive disorders of pregnancy, in whom only 21 EPDS-positive events were observed. Therefore, further studies with larger sample sizes are needed to confirm these findings.

This study has several limitations. First, its retrospective, single-center design limits causal inference and generalizability. Second, postpartum depressive symptoms were identified using EPDS screening rather than a structured psychiatric interview; therefore, the outcome represents screening-positive symptom status rather than a clinical diagnosis of depressive disorder. Although EPDS item 10 responses were available, detailed information regarding subsequent clinical risk assessment, psychiatric referral, or follow-up management was not separately extracted from the retrospective dataset. Third, although prenatal PHQ-9 and GAD-7 scores were available, family support, major life events, perceived stress, physical activity, sunlight exposure, dietary vitamin D intake, and adherence to vitamin D supplementation were not fully captured. Fourth, serum 25(OH)D was measured only once during early pregnancy, precluding assessment of longitudinal changes. Fifth, complete baseline variables were unavailable for most excluded women, and a formal comparison between included and excluded participants could not be performed. Finally, only 69 outcome events were observed, and several exploratory subgroups contained few events, reducing statistical precision and increasing susceptibility to influential observations.

Direct reverse causation from postpartum symptoms to first-trimester vitamin D status is temporally unlikely because vitamin D was measured before the postpartum outcome. However, prenatal psychological symptoms and shared behavioral determinants may influence both first-trimester vitamin D status and subsequent postpartum EPDS scores. Future prospective multicenter studies should therefore incorporate repeated measurements of vitamin D and psychological symptoms, detailed assessment of psychosocial and lifestyle factors, and standardized psychiatric evaluation. Such studies are needed to determine whether vitamin D provides information beyond established prenatal psychological and clinical factors.

## Conclusion

Lower first-trimester serum 25(OH)D was associated with EPDS-positive postpartum depressive symptoms in crude and baseline-adjusted analyses among women with GDM, but the association was substantially attenuated and became statistically non-significant after adjustment for prenatal depressive and anxiety symptoms. Fully adjusted stratified analyses did not identify statistically significant associations or effect modification according to pre-pregnancy BMI, hypertensive disorders of pregnancy, or insulin use. These findings highlight the importance of accounting for prenatal mental health when evaluating the relationship between maternal vitamin D status and postpartum psychological outcomes.

## Data Availability

The raw data supporting the conclusions of this article will be made available by the authors, without undue reservation.
